# Intranasal parainfluenza virus type 5 (PIV5)–vectored RSV vaccine is safe and immunogenic in healthy adults in a phase 1 clinical study

**DOI:** 10.1126/sciadv.adj7611

**Published:** 2023-10-25

**Authors:** Paul Spearman, Hong Jin, Kristeene Knopp, Peng Xiao, Maria Cristina Gingerich, Jamie Kidd, Karnail Singh, Marinka Tellier, Henry Radziewicz, Samuel Wu, Matthew McGregor, Barbara Freda, Zhaoti Wang, Susan P. John, Francois J. Villinger, Biao He

**Affiliations:** ^1^ Department of Pediatrics, Cincinnati Children’s Hospital, 3333 Burnet Avenue, Cincinnati, OH 45229, USA.; ^2^ Department of Pediatrics, University of Cincinnati College of Medicine, 3333 Burnet Avenue, Cincinnati, OH 45229, USA.; ^3^ Blue Lake Biotechnology Inc., 111 Riverbend Rd., Athens, GA 30602, USA.; ^4^ New Iberia Research Center, University of Louisiana at Lafayette, New Iberia, LA 70560, USA.

## Abstract

Respiratory syncytial virus (RSV) can lead to serious disease in infants, and no approved RSV vaccine is available for infants. This first in-human clinical trial evaluated a single dose of BLB201, a PIV5-vectored RSV vaccine administrated via intranasal route, for safety and immunogenicity in RSV-seropositive healthy adults (33 to 75 years old). No severe adverse events (SAEs) were reported. Solicited local and systemic AEs were reported by <50% of participants and were mostly mild in intensity. Vaccine virus shedding was detected in 17% of participants. Nasal RSV-specific immunoglobulin A responses were detected in 48%, the highest level observed in adults among all intranasal RSV vaccines evaluated in humans. RSV-neutralizing antibodies titers in serum rose ≥1.5-fold. Peripheral blood RSV F–specific CD4^+^ and CD8^+^ T cells increased from ≤0.06% at baseline to ≥0.26 and 0.4% after vaccination, respectively, in >93% participants. The safety and immunogenicity profile of BLB201 in RSV-seropositive adults supports the further clinical development of BLB201.

## Teaser

Intranasal RSV vaccine based on kennel cough vaccine component PIV5 is safe and immunogenic in humans.

## INTRODUCTION

Respiratory syncytial virus (RSV) is the leading global cause of morbidity and mortality in infants due to infection-associated bronchiolitis and pneumonia, especially in those with underlying cardiopulmonary disease (*1*–*3*). The global yearly incidence of RSV-associated acute respiratory tract infections is estimated at 33 million children aged <5 years, leading to 3 million hospitalizations and up to 60 to 200 thousand deaths (*4*). RSV also causes substantial disease burden in the elderly (*5*, *6*). In the United States, RSV is responsible for approximately 60 to 160 thousand hospitalizations and 6 to 13 thousand deaths annually in adults ≥65 years of age (*7*, *8*). The high burden of disease caused by RSV and its economic costs makes RSV a high priority target for vaccine development.

RSV causes both upper and lower respiratory diseases. Recently, RSV prefusion F (preF) protein–based vaccines have been shown to be effective in reducing symptoms of RSV-associated lower respiratory tract disease in elderly (aged ≥60 years) in clinical trials and received approval for elderly (*9*–*12*). Still, there is no RSV vaccine that prevents upper and/or lower respiratory tract infection/diseases. Furthermore, the clinical development of RSV vaccines for infants and young children has not led to a vaccine product yet. Because of concerns for enhanced RSV diseases in children with inactivated RSV vaccine, those development programs have primarily used intranasally delivered live-attenuated RSV vaccines and viral-vectored vaccines (*13*). While immune correlates of protection for RSV infection are not clear, nasal antibody (Ab), especially nasal RSV-specific immunoglobulin A (IgA), is thought to be critical for protection against RSV infection (*14*), leading to the focus on intranasal approaches for RSV vaccine development, especially for infants and children. In clinical evaluations in phase 1 and 2 trials in infants who were RSV naïve (RSV seronegative), some of the attenuated RSV vaccines have been shown to promote RSV-specific Ab and cell-mediated immunity (CMI) (*13*, *15*–*17*). Although the safety profiles of these vaccines have been generally acceptable, the challenge with the live-attenuated RSV vaccines has been to identify a favorable balance between immunogenicity and safety (*13*, *15*). Additional hurdle for attenuated RSV vaccine includes maternal anti-RSV Ab and long-acting RSV-neutralizing antibodies (nAbs) that have been approved for use in infants. A bovine parainfluenza virus 3 (bPIV3)–vectored RSV vaccine was evaluated in adults and infants (*18*, *19*) but was abandoned because of the genetic instability of the vaccine. An intranasal chimpanzee adenovirus (ChAd)–vectored vaccine, PanAd3-RSV, has also been evaluated in adults as part of a prime-boost regimen in combination with intramuscular vaccination (*20*, *21*). A single dose of intranasal PanAd3-RSV immunization was well tolerated in humans but did not generate detectable immune responses to RSV in adults.

BLB201 is a live viral-vectored candidate RSV vaccine based on a parainfluenza virus 5 (PIV5) encoding the RSV F antigen. PIV5 is a negative-sense RNA virus and replicates in the cytoplasm of infected cells without a DNA phase in its life cycle. PIV5 is a component of the kennel cough vaccine commonly administered by intranasal route in dogs. Vaccinated dogs can shed virus up to 5 days after dosing, exposing humans to PIV5. However, although the vaccine has been used for 50 years, no known human illness has been reported from human exposure to vaccinated dogs (*22*). PIV5 infects many cell types using sialic acid receptors with minimal cytopathic effect, allowing it to infect mucosal cells in vivo. Intranasal PIV5-vectored vaccines have been effective against respiratory pathogens including influenza viruses, Middle East respiratory syndrome coronavirus, and severe acute respiratory syndrome coronavirus 2 in animal models via intranasal immunization (*23*–*29*). In preclinical studies, a single intranasal dose of PIV5-vectored RSV vaccine induced RSV F–specific Ab and CMI in mice, cotton rats, and African green monkeys and protected against RSV challenge (*30*–*32*). Here, we report the first in-human clinical study of PIV5-vectored RSV vaccine BLB201 in RSV-seropositive healthy adults in the United States.

## RESULTS

### Participants and study conduct

A total of 30 subjects were enrolled in this phase 1 trial, and all received a single intranasal dose of BLB201 at 10^7.5^ plaque forming units (PFU). The vaccine was administrated as a 0.25-ml spray to each nostril (total volume of 0.5 ml) using a MAD Nasal Intranasal Mucosal Atomization Device (Teleflex MAD300). The mean ages in group 1 (planned participant ages 18 to 59 years and actual enrollee ages 33 to 59 years) and group 2 (planned participant ages 60 to 75 years and actual enrollee ages 61 to 75 years) were 45 and 67 years, respectively (Table 1). The majority of participants were female (70%). Most participants in the two groups were white (73 and 80% in groups 1 and 2, respectively). All except one participant completed the study, and this participant in group 1 was lost to follow-up after day 7.

**Table 1.** Trial cohort demographics.

Group	Group 1	Group 2
*N*	15*	15
Mean age (range)	44.9 (33, 59)	66.8 (61, 75)
Male/female	4/11	5/10
Race/ethnicity	11 white (73%), others-Black/African American and American Indian	12 white (80%), others-Black/African American and Asian

*One participant dropped out of the study on day 8 (lost to follow-up).

### Safety

After intranasal immunization with Teleflex MAD300, the safety and tolerability of BLB201 were monitored for 6 months. Participants recorded information of local and systemic adverse events (AEs) for 7 days after vaccination (solicited local and systematic events) and unsolicited AEs for 28 days using a memory aid. No serious AE (SAE) assessed as related to vaccine was reported, and no symptomatic RSV infection was reported during the 6-month trial period. In the 1-week period after vaccination, all solicited local AEs that were reported were mild in intensity (grade 1; Table 2). Runny nose was the most common reaction (in 7 of 15 participants in each age group). Sore throat and cough were reported by 4 of 15 and 3 of 15 participants, respectively, in group 1 and by 1 of 15 participants for each symptom in group 2. Within 1 hour after vaccination, post-nasal drip was reported by four participants and difficulty in concentration by one participant.

**Table 2.** Summary of local and systemic reactogenicity and AEs. Grading scale: Grade 1 = mild (awareness of a symptom but the symptom is easily tolerated); grade 2 = moderate (discomfort enough to cause interference with usual activity); grade 3 = severe (incapacitating; unable to perform usual activities; requires absenteeism from work or bed rest). AE, adverse event.

Group	Group 1	Group 2	
Solicited local AE (days 1–8): Number (and percentage) of subjects reporting grade 1 events (no grade 2 or grade 3 events reported)			
	Grade 1	Grade 1	
Runny nose/nasal congestion	7 (47%)	7 (47%)	
Sore throat	4 (27%)	1 (7%)	
Cough	3 (20%)	1 (7%)	
Solicited systematic AE (days 1–8): Proportion of subjects reporting grades 1 and 2 (no grade 3 events reported). Only grade 1 AE reported in group 1.			
	Grade 1	All Grades	Grade 2
Fever	0	0	0
Headache	2 (13%)	5 (33%)	3 (20%)
Myalgia	1 (7%)	5 (33%)	2 (13%)
Fatigue	5 (33%)	2 (13%)	1 (7%)
Chills	0	1 (7%)	0
Nausea/Vomiting	0	1 (7%)	0
Breathing discomfort	0	1 (7%)	0
Unsolicited AE (assessed as possibly related to vaccine): Only grade 1 AEs reported.			
Difficult concentration for 1 hour after dosing	1 (7%)	0	
Post-nasal drip after dosing	4 (27%)	0	
Brain fog	0	1 (7%)	

In group 1, all solicited systemic AEs reported by 5 of 15 participants were mild in intensity and included fatigue, headache, and myalgia being reported by 5 of 15, 2 of 15, and 1 of 15 participants, respectively. In group 2, the solicited systemic AEs reported by 8 of 15 participants were mild or moderate in intensity and included fatigue, headache, and myalgia being reported by 2 of 15, 5 of 15, and 5 of 15 participants, respectively, as well as chills, nausea/vomiting, and breathing discomfort being reported by 1 of 15 for each symptom. The single episode of breathing discomfort was mild and resolved within 2 days after vaccination. No solicited fever was reported in either group. Throughout the 6-month study period, five medically attended AEs were reported by four participants (all in group 2), none of which was assessed as being related to study vaccine.

### Vaccine virus shedding and genetic stability

**Fig. 1.**
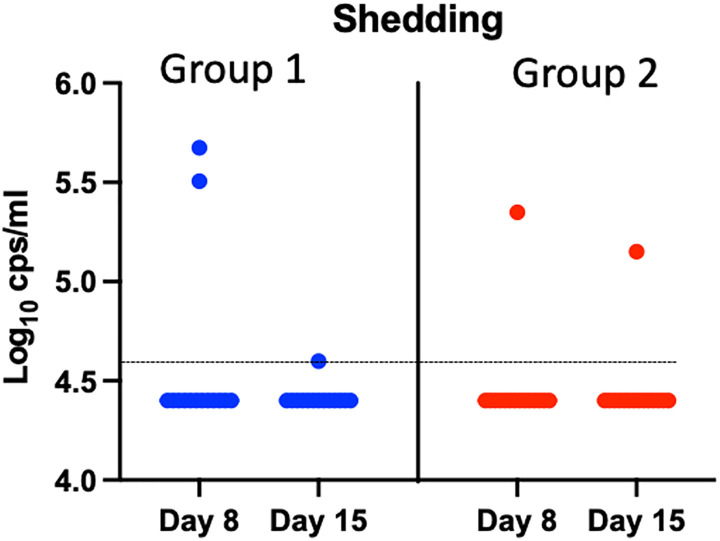
Vaccine-virus shedding. PIV5 viral genomic RNA detection by RTqPCR in individual BLB201 recipients at days 8 and 15. The dotted line indicates the LOD at 4.6 log_10_ copies/ml.

At day 1 before participants were immunized, no vaccine virus genomic RNA was detected as expected [i.e., below the lower limit of detection (LOD) by reverse transcription quantitative polymerase chain reaction (RT-qPCR)] in the nasal swabs of any participant (Fig. 1). At either 1 or 2 weeks after vaccination, the vaccine virus genomic RNA was detected in the nasal swabs of five participants: three participants in group 1 and two participants in group 2. For two participants in which vaccine virus genomic RNA was detected at 2 weeks after vaccination, the vaccine vector was no longer detected at 4 or 8 weeks after vaccination. The sequences of the BLB201 were successfully obtained in four of five positive samples, and they matched the original sequence for the inserted RSV F gene and the flanking regions. This suggested that BLB201 remained genetically stable after vaccination, and shedding of the vaccine virus lasted <4 weeks after vaccination.

### Immunogenicity

#### Serum Ab titers

**Fig. 2.**
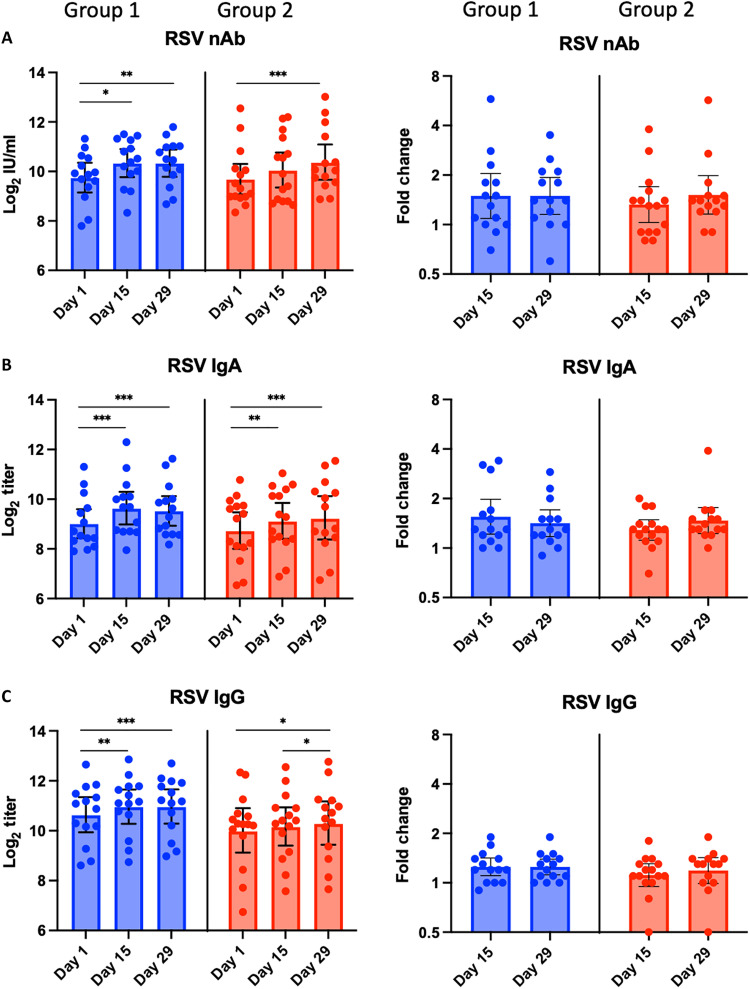
Serum RSV-specific Ab titers before and after vaccination. (**A**) RSV nAbs by microneutralization assay and (**B**) F-specific RSV IgA Abs and (**C**) F-specific IgG Abs by enzyme-linked immunosorbent assay (ELISA). Individual log_2_ titers and mean log_2_ titers with the respective SDs (error bars) at days 1, 15, and 29 by age group are shown in the graphs in the left column, and GMFRs from baseline on days 15 and 29 by age group are shown in the graphs in the right column. *P < 0.05, **P < 0.01, and ***P < 0.001 by Wilcoxon test.

All participants were seropositive for RSV nAbs at baseline, and the titer changes are presented in Fig. 2 and fig. S1A. In group 1, the nAb geometric mean titer (GMT) was 880 (9.8 log_2_) at baseline and increased to 1316 (10.4 log_2_) at 2 weeks and 1312 (10.4 log_2_) at 4 weeks after vaccination (*P* < 0.05), reflecting a geometric mean fold rise (GMFR) of 1.5. RSV nAb seroresponses (≥1.5-fold rise) were identified in 7 of 14 (50%) participants. In group 2, the nAb GMT was 850 (9.7 log_2_) at baseline, similar to that in group 1, and increased to 1103 (10.1 log_2_) at 2 weeks (*P* < 0.05) and 1372 (10.4 log_2_) at 4 weeks after vaccination (*P* < 0.05), reflecting GMFRs of 1.3 and 1.5, respectively. RSV nAb seroresponses were identified in 6 of 15 (40%) participants in this group (Table 3) (individual nAb titer changes were shown in fig. S1A). Notably, RSV nAb seroresponses were identified in all five participants positive for vaccine virus shedding, suggesting a potential correlation between replication and systemic Ab responses.

**Table 3.** Summary of RSV Ab and CMI response rates. A positive seroresponse was defined as ≥1.5-fold rise from baseline for RSV F IgG and IgG. A positive nasal IgA Ab response was defined as ≥2-fold rise over baseline. CMI response was defined as >0.1% rise of sum of individual T cell secreting >1 cytokine after subtraction of baseline (before vaccination). CTL response was defined by the change in IFN-γ–producing CD8^+^ cells. CMI, cell-mediated immunity; CTL, cytotoxic T lymphocyte; PBMC, peripheral blood mononuclear cell; PIV5, parainfluenza virus type 5; RSV, respiratory syncytial virus.

Sample	Immune parameter	Group 1 responders	Group 2 responders
		*n*/*N* (% responder)	*n*/*N* (% responder)
Serum	RSV nAb	7/14 (50%)	6/15 (40%)
	RSV F IgA	6/14 (43%)	5/15 (36%)
	RSV F IgG	3/14 (21%)	1/15 (7%)
	PIV5 nAb	7/14 (50%)	10/15 (67%)
	PIV5 IgG	12/14 (86%)	13/15 (87%)
Nasal swab	Nasal RSV F IgA	9/14 (64%)	5/15 (33%)
PBMC	Total CMI	13/14 (93%)	15/15 (100%)
	CD8 CTL	12/14 (86%)	13/15 (87%)

All participants were also seropositive for RSV F–specific serum IgA and IgG Abs at baseline (Fig. 2). In froup 1, F-specific serum IgA GMT increased from 530 (9.0 log_2_) at baseline to 821 (9.7 log_2_) and 756 (9.6 log_2_) at 2 and 4 weeks after vaccination, respectively. F-specific serum IgG GMT increased from 1640 (10.7 log_2_) at baseline to 2049 (11.0 log_2_) at 2 weeks and 2055 (11.0 log_2_) at 4 weeks after vaccination. F-specific serum IgA and IgG seroresponses (≥1.5-fold) were identified in 6 of 14 (43%) and 3 of 14 (21%) participants, respectively (Table 3). In group 2, F-specific serum IgA GMT increased from 445 (8.8 log_2_) at baseline to 582 (9.2 log_2_) and 641 (9.3 log_2_) at 2 and 4 weeks after vaccination, respectively. F-specific serum IgG GMT increased from 1088 (10.1 log_2_) at baseline to 1198 (10.2 log_2_) and 1325 (10.4 log_2_) at 2 and 4 weeks after vaccination. F-specific IgA and IgG seroresponses were identified in 5 of 15 (33%) and 1 of 15 (7%) participants, respectively (Table 3).

Overall, the results suggested that BLB201 boosted RSV-specific serum Ab levels in young adults (33 to 59 years old) and elderly (61 to 75 years old), although with greater magnitude in adults versus elderly. The fold increases in RSV-specific Ab titers inversely correlated with baseline titers. It is possible that the higher baseline titers were close to maximum levels.

**Fig. 3.**
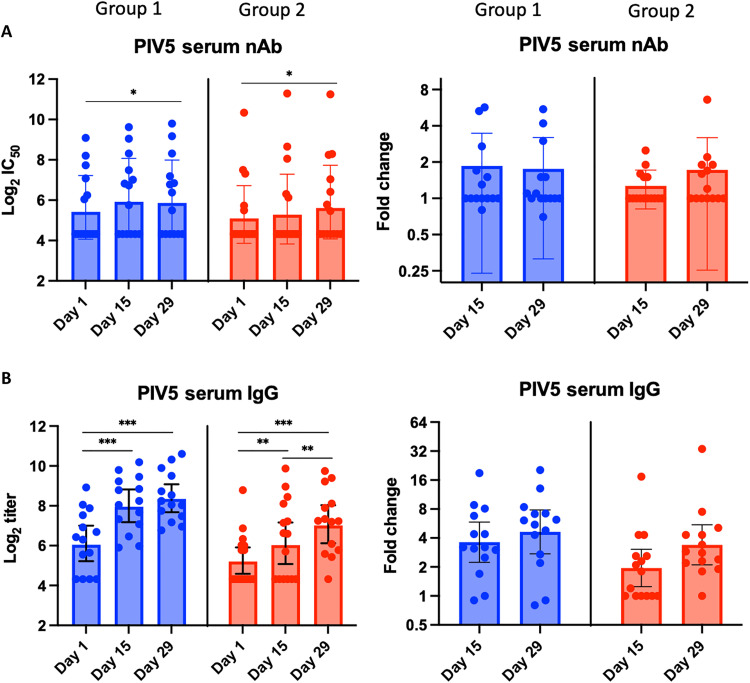
Serum PIV5 Ab titers before and after vaccination. (**A**) PIV5 nAbs by microneutralization assay and (**B**) PIV5-specific RSV IgG Abs by ELISA. Individual log_2_ titers, and mean log_2_ titers with the respective SDs (error bars) at days 1, 15, and 29 by age group are shown in the graphs in the left column, and GMFRs from baseline on days 15 and 29 by age group are shown in the graphs in the right column. *P < 0.05, **P < 0.01, and ***P < 0.001 by Wilcoxon test.

We next examined vector-specific immune responses. For PIV5 nAbs at baseline, 15 of 29 (52%) participants were seropositive as defined by a titer of 1:10 (Fig. 3A). This level of background seropositivity likely reflects passive exposure to PIV5 through exposure to dogs receiving the kennel cough vaccine. In group 1, the PIV5 nAb GMT increased from 26 (4.7 log_2_) at baseline to 48 (5.6 log_2_) and 51 (5.7 log_2_) at 2 and 4 weeks after vaccination, respectively, reflecting a GMFRs of 1.8 and 2.0, respectively. PIV5 nAb seroresponses (≥1.5-fold rise) were identified in 7 of 14 (50%) participants (Table 3). In group 2, the PIV5 nAb GMT increased from 19 (4.2 log_2_) at baseline to 34 (5.1 log_2_) and 44 (5.5 log_2_) at 2 and 4 weeks after vaccination, respectively, reflecting a GMFRs of 1.8 and 2.2, respectively. PIV5 nAb seroresponses (≥1.5-fold rise) were identified in 10 of 15 (67%) participants. Similar kinetics were observed with PIV5-specific IgG titers (Fig. 3B) but with greater GMFRs (≤4.6-fold in group 1 and ≤3.4-fold in group 2) and seroresponses (86% in group 1 and 87% in group 2). By comparing participants with low PIV5 nAb titers with those with high PIV5 nAb titers, baseline PIV5 nAb titers had no clear effect on PIV5 nAb seroresponse rate and appeared to not inhibit RSV nAb seroresponse rate (fig. S2).

#### RSV F–specific IgA Ab titers in nasal swabs

**Fig. 4.**
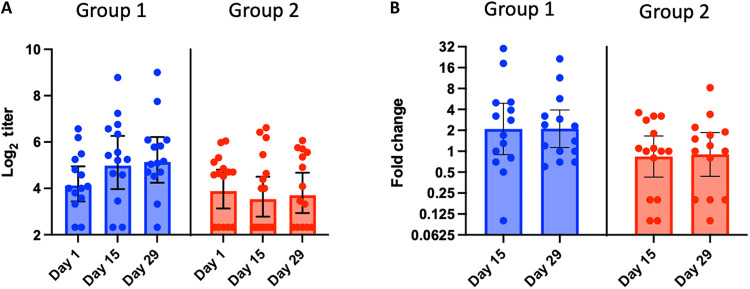
Nasal IgA Ab titers before and after vaccination. F-specific RSV IgA Abs by ELISA. Individual log_2_ titers, and mean log_2_ titers with the respective SDs (error bars) at days 1, 15, and 29 by age group are shown in the graphs in (**A**), and GMFRs from baseline on days 15 and 29 by age group are shown in the graphs in (**B**).

At baseline, RSV F–specific IgA Ab titers in nasal swabs were variable ranging from below the LOD to 514 (9.0 log_2_) (Fig. 4A). In group 1, the F-specific nasal IgA GMT was 19 (4.2 log_2_) at baseline and increased to 40 (5.3 log_2_) at 2 and 4 weeks after vaccination (*P* < 0.05), reflecting a GMFR of 2.1 (Fig. 4B). F-specific nasal IgA responses (≥2-fold rise) were identified in 9 of 14 (64%) participants (Table 3). In group 2, the baseline F-specific nasal IgA GMT of 18 (4.2 log_2_) was similar to that in group 1, but GMTs were not boosted (*P* > 0.05) 2 and 4 weeks after vaccination (Fig. 4, A and B). However, F-specific nasal IgA responses were identified in 5 of 15 (33%) participants in group 2 (individual’s nasal IgA Ab changes were shown in fig. S1B). In groups 1 and 2, F-specific nasal IgA responses were identified in four of five participants positive for vaccine virus shedding.

Overall, the results suggested that BLB201 boosted RSV-specific nasal IgA levels in adults and elderly but to a lesser degree in elderly. Similar to results seen with systemic Ab responses, the fold increases in F-specific nasal IgA titers in both groups inversely correlated with baseline titers (fig. S2).

#### Cell mediated immunity

**Fig. 5.**
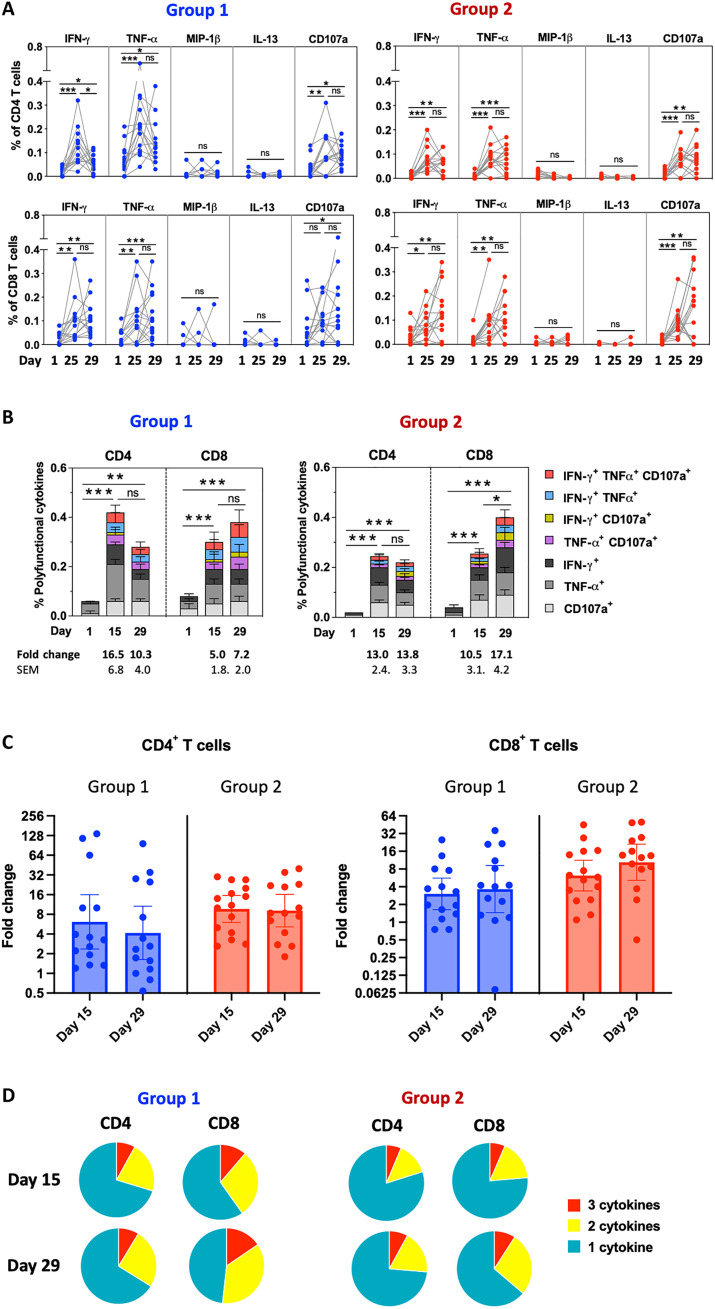
RSV F–specific CD4^+^ and CD8^+^ T cell frequencies per million PBMCs (by percentage) before and after vaccination. (**A**) Percentages of CD4^+^ T cells (top graphs) and CD8^+^ T cells (bottom graphs) by age group on days 1, 15, and 29 for each individual (represented by symbols and contiguous line) by expression of the indicated immune marker. (**B**) Mean percentages of CD4^+^ T cells (left graphs) and CD8^+^ T cells (right graphs) by age group on days 1, 15, and 29 by expression of the indicated T_H_1/cytotoxic marker combinations (IFN-γ, TNF-α, and CD107a) represented in stacked histograms. (**C**) GMFRs from baseline on days 15 and 29 by age group for CD4^+^ and CD8^+^ T cells. (**D**) Pie chart representations of the proportions of F-specific CD4^+^ and CD8^+^ T cells expressing one, two, or three T_H_1/cytotoxic markers (IFN-γ, TNF-α, and CD107a) at days 15 and 29 in groups 1 and 2. *P < 0.05, **P < 0.01, and ***P < 0.001. ns, not significant.

At baseline, RSV F–specific CD4^+^ and CD8^+^ T cells were detected on the basis of the expression of T helper 1 (T_H_1) or cytotoxic T cell cytokines/markers interferon-γ (IFN-γ), tumor necrosis factor–α (TNF-α), macrophage inflammatory protein–1β (MIP-1β), and CD107a and on the expression of T_H_2 cytokine interleukin-13 (IL-13) (Fig. 5A). In group 1, the mean percentages of F-specific CD4^+^ T cells expressing at least one of the T_H_1/cytotoxic markers increased from 0.06% at baseline to a maximum of 0.42% 2 weeks after vaccination (*P* < 0.001; Fig. 5B), and the mean percentages of the same phenotypes of CD8^+^ T cells increased from 0.08% at baseline to a maximum of 0.38% 4 weeks after vaccination (*P* < 0.001). In group 2, the mean percentages of the same phenotypes of CD4^+^ T cells increased from 0.02 to a maximum of 0.26% 2 weeks after vaccination (*P* < 0.001), and CD8^+^ T cells increased from 0.04 to a maximum of 0.40% 4 weeks after vaccination (*P* < 0.001). In groups 1 and 2, F-specific CMI responses to BLB201 (defined as an increase from baseline of >0.1% of T cells expressing at least two T_H_1/cytotoxic biomarkers) were identified in 13 of 14 (93%) and 15 of 15 (100%) participants, respectively (Table 3). Also, F-specific cytotoxic T lymphocyte (CTL) responses to BLB201 (defined as an increase from baseline of >0.1% of CD8^+^ T cells expressing IFN-γ) were identified in 12 of 14 (86%) and 13 of 15 (87%) participants, respectively. This suggested that BLB201 enhanced the production of F-specific T_H_1 (CD4^+^) and cytotoxic (CD8^+^) T cells in nearly all participants. Although numbers of CD4^+^ and CD8^+^ T cells were similar after immunization in both groups, the geometric mean of CD4^+^ T cells rose to 6.1- and 9.1-fold by day 15 for groups 1 and 2, respectively, and the geometric mean of CD8^+^ T cells rose to 3.6- and 10-fold by day 29 for groups 1 and 2, respectively (Fig. 5C), reflecting lower baseline for group 2 than for group 1. This result suggests that, in contrast to nasal IgA response, similar levels of CMI in adults and elderly was induced by BLB201.

In both groups 1 and 2, T cells expressing a single T_H_1/cytotoxic biomarker were more frequent than those expressing at least two T_H_1/cytotoxic markers (Fig. 5D). However, T cells coexpressing at least two T_H_1/cytotoxic biomarkers tended to be more frequent in group 1 versus group 2, suggesting that BLB201 may induce better quality of antigen-specific effector/memory T cells in the younger age group based on polyfunctional T_H_1/cytotoxic responses. In contrast to T_H_1 and cytotoxic phenotypes, the percentages of F-specific CD4^+^ T cells and CD8^+^ T cells expressing IL-13 did not increase after vaccination in either group 1 or group 2 (Fig. 5A), indicating that there was no T_H_2-biased response to BLB201, which has been identified as a potential risk factor for vaccine-associated enhanced RSV disease (*33*, *34*).

## DISCUSSION

This clinical study of the PIV5-vectored RSV vaccine candidate BLB201 was the first in-human study for this vaccine and for the replicating PIV5 vector. The study showed that BLB201 administered as a single intranasal dose had an acceptable safety profile. Solicited local and systemic AEs were generally mild and only reported by approximately half of the vaccine recipients. No SAE related to vaccination was reported. This result is consistent with the long history of PIV5 vector’s use in dogs. The vector has been used in dogs for 50 years, and yet no human disease has ever been reported from human exposure to PIV5-immunized animals. In this phase 1 study, approximately half of the enrolled participants were PIV5 seropositive based on nAb titer >1:10 at baseline, suggesting that asymptomatic exposures occur in a high percentage of the U.S. population. The safety profile of BLB201 is comparable to other intranasal live virus–based vaccines including live-attenuated RSV vaccines, MEDI-534, and PanAd3-RSV in adults (*18*, *20*, *21*). The acceptable safety profile of PIV5 vector in this first in-human trial will enable the development of other vaccines for humans using PIV5 vector platform.

Although RSV preF protein–based vaccines have been approved for reducing symptoms in lower respiratory tract of RSV-infected elderly, an RSV vaccine that prevents symptomatic infection in lower respiratory tract and even symptomatic infection in upper respiratory tract is highly desirable. While it is thought that serum Ab, nasal IgA, and CMI all contribute to protection against symptomatic RSV infection, RSV-specific nasal IgA is thought to be the most critical in preventing RSV infection (*35*, *36*). While high levels of serum nAb against RSV can reduce severity of RSV diseases, data for demonstrating that serum nAb can prevent RSV infection has been inconsistent. It was reported that higher RSV-specific nasal IgA level correlates with lower level of RSV infection-associated clinical symptoms in human challenge studies with wild-type virus (*37*). Furthermore, lower level of RSV-specific nasal IgA is a risk factor for RSV infection (*17*). Thus, generating RSV-specific nasal IgA is a viable approach to develop an RSV vaccine that can prevent RSV infection. CMI plays a critical role in virus clearance. However, the study of CMI in RSV immunity is limited. BLB201 was immunogenic in seropositive adults, even in those with pre-existing antivector immunity. BLB201 induced a range of immune responses, from the enhanced production of RSV F–specific Ab in the nasal mucosa to enhanced RSV nAbs and cytotoxic T cells in the peripheral blood.

There are several intranasal RSV vaccines advanced to human clinical trials. Most are live-attenuated RSV vaccines. Because of the existing anti-RSV antibodies, these live-attenuated RSV vaccines did not generate detectable immune responses in RSV-seropositive people (*38*). Viral vectored RSV vaccines such as MEDI-534 (b/hPIV3-based RSV), Sendai virus–vectored RSV vaccine and ChAd-RSV did not generate measurable immune responses in adults after a single dose of intranasal immunization (*18*, *20*, *21*). Unlike these intranasal RSV vaccines that failed to generate detectable vaccine-mediated immune responses in RSV-seropositive adults, an increase in both serum RSV nAbs and nasal F-specific IgA Abs has been observed for BLB201 intranasal vaccine in adults. BLB201 induced nasal IgA in both age groups, albeit at a higher frequency in the younger age group. We posit that the more robust immune responses generated by BLB201 is likely due to the fact that BLB201 replication was detected in human adults in this clinical trial. The mucosal immunity elicited by BLB201 has the potential to limit virus replication by reducing RSV infection at the portal of viral entry, an important protection mechanism as a prophylactic vaccine. To the best of our knowledge, BLB201 generates the most robust RSV F–specific nasal IgA response among all RSV vaccine candidates.

The induction of cytotoxic CD8^+^ T cells by BLB201 was observed in the majority of participants in this study. Notably, cytotoxic CD8^+^ T cell induction is often minimally induced by parenteral RSV subunit vaccine (*21*, *39*). Cytotoxic CD8^+^ T cells are considered essential effectors that eliminate infected cells, enabling viral clearance (*40*). Polyfunctional T cells that have been associated with protection against disease (*41*) were also induced by BLB201, while no T_H_2-biased T cell responses to RSV were induced in this study; T_H_2 type of responses are considered a risk factor for vaccine-enhanced RSV disease (*33*, *34*). The lack of T_H_2-biased responses from BLB201 immunization further supports the BLB201 safety profile. While the precise role of CD8^+^ CTL in preventing RSV infection and disease is not well defined, it is probable that high levels of CD8^+^ CTL induced by BLB201 can play a positive role in immunity against RSV disease. Expectedly, base levels of CD8^+^ in group 1 (33 to 59) with younger participants (0.08%) is a little higher than group 2 (61 to 75) with elderly (0.04%). Both groups reached similar levels of CD8^+^ at 0.38 and 0.40% after BLB201 immunization, indicating that BLB201 can induce robust CD8^+^ CTL in elderly (group 2).

The rate of the induction of nasal F-specific IgA Ab response was higher in the younger age group than the elderly group, indicating that, similar to many other vaccines, there was an age-dependent difference in magnitude for responses to BLB201 in nasal IgA Ab. This may be attributed to age-related immunosenescence (*42*), suggesting that a higher dosage or a second dose may be required in the older population. Because BLB201 generated similar CMI but different levels of nasal IgA in elderly, investigating the efficacy of BLB201 in elderly may elucidate the roles of CD8^+^ CTL in preventing RSV-associated diseases in elderly.

In this study, approximately 50% (group 1) and 40% (group 2) of participants were seropositive at baseline for PIV5 nAbs. Seropositivity to PIV5 in this study was a little higher than previously reported at about 30% (*43*), potentially due to prior exposure to dogs vaccinated with PIV5-containing live kennel cough vaccine. In this study, pre-existing PIV5-specific Ab titers did not appear to negatively affect RSV-specific immune responses to BLB201, suggesting that immune experience to PIV5 had no or limited impact on the ability to elicit RSV-specific responses to BLB201. This result is consistent with our previous report that PIV5-vectored influenza vaccine generated similar immune responses in PIV5 immune naïve dogs and in kennel cough vaccinated dogs, which might be due to its ability to infect multiple mucosal cell types in the presence of serum PIV5 nAb (*43*).

The shedding of vaccine virus in nasal swabs after vaccination was transient, lasting less than 4 weeks, suggesting that BLB201 has the capacity to replicate in the nasal cavities of RSV-experienced adults. This self-limiting infection in the respiratory mucosa by BLB201 may help in the development of RSV-specific immunity. Although vaccine virus shedding was only identified in five participants, it is plausible that it had occurred in other participants but at earlier time points than were sampled here, i.e., within 1 week after vaccination. The result that over 93% participants had robust anti-RSV F cellular immune responses is consistent with replication of BLB201 in humans: BLB201 likely entered human cells and expressed RSV F that was presented by major histocompatibility complex I in infected cells.

The genetic stability of the viral vaccine vector is important for its use in humans. MEDI-534 was not stable in infants, resulting in the termination of its clinical development (*19*, *44*). Extensive testing of BLB201 genetic stability in vitro, in animals, and in this study has indicated that BLB201 is genetically stable in RSV-seropositive adults (*31*).

In conclusion, BLB201 is a PIV5-based intranasal RSV candidate vaccine that demonstrated safety and immunogenicity in this phase 1 study. The ability of BLB201 to boost pre-existing cellular, humoral, and mucosal responses in adults supports further clinical evaluation of this approach in infants and adult populations.

## MATERIALS AND METHODS

### Study design and vaccination

The phase 1 clinical trial of PIV5-RSV vaccine (BLB201; ClinicalTrials.gov, NCT05281263) was approved by the Advarra central Institutional Review Board (IRB) and conducted at two study sites in the United States. Participants were recruited to two study cohorts, healthy young adults (group 1, 33 to 59 years old) and healthy older adults (group 2, 61 to 75 years old). Participants of childbearing potential were required to practice contraceptive measurement to prevent pregnancy. Exclusion criteria included any live vaccine within the 30 days before trial vaccine; any prior receipt of any investigational RSV vaccine or any PIV5-based vaccine (CVXGA1) that was actively enrolling during the trial period; and known infection with human immunodeficiency virus, hepatitis B virus, or hepatitis C virus. A full list of inclusion and exclusion criteria can be found at https://clinicaltrials.gov. Participants were not prescreened for their RSV serum Ab levels. Eligible participants were administered on day 1, a single dose at a concentration of 10^7.5^ PFU of BLB201 and a 0.25-ml spray to each nostril (total volume 0.5 ml) using a MAD Nasal Intranasal Mucosal Atomization Device (Teleflex MAD300), and observed for 30 min immediately after dosing. In addition, participants were asked to maintain a memory aid for solicited systemic AEs and local reactions during the week after vaccination. Four sentinel participants were dosed first in each group, and their safety data were reviewed by the safety monitory committee before the remainder of the participants in the group were enrolled.

Primary outcome measures included (i) solicited AEs (days 1 to 8) and (ii) unsolicited AEs (days 1 to 29). Secondary outcome measures included (i) serum IgG titers to RSV protein (days 15 and 29), (ii) SAEs (days 1 to 181), and (iii) AEs of special interest (AESIs) including new onset chronic medical conditions and medically attended AEs (days 1 to181).

### Safety assessments

The safety and tolerability of PIV5-RSV intranasal vaccine were assessed over the period of 6 months after vaccination. Participants recorded the information of local and systemic AEs for 7 days after vaccination (solicited local and systematic events) and unsolicited AEs for 28 days using a memory aid. SAEs and AESIs were collected at scheduled study follow-up visits or unscheduled visits for the full duration of the study (6 months). AEs were graded according to the U.S. Food and Drug Administration Center for Biologics Evaluation and Research Toxicity Grading Scale for Healthy Adult and Adolescent Volunteers Enrolled in Preventive Vaccine Clinical Trials guidelines. All unsolicited AEs were categorized by the investigator for possible causative association with vaccination (i.e., relatedness to vaccination).

### Vaccine shedding and genetic stability

Vaccine-virus shedding was analyzed by RT-qPCR of nasal swabs collected on days 1 (before vaccination) and days 8, 15, and 29 after vaccination. A regular sized flocked nylon swab was used to collect nasal secretion from one nostril, placed in 1-ml universal transport medium (VTM, Copan), and stored at −80°C until sample testing. RNA was extracted from 140 μl (group 2) or 560 μl (group 1) of nasal swab by the QIAmp Viral RNA Mini Kit (QIAGEN) and eluted in 40 μl of buffer. The primers targeted the trailer region of PIV5 genome to quantify PIV5-RSV vaccine virus genomic RNA. The PCR reaction mix included Luna Universal probe One-Step RT-qPCR (New England BioLabs), PIV5 trailer region primers/probe (sequence information is available upon request), and 8 μl of RNA in a QuantStudio 3 instrument (Thermo Fisher Scientific). The assay had a lower LOD of 4.6 log_10_ copies/ml (~2.5 log_10_ PFU/ml) and a linearity from 4.6 to 9.0 log_10_ copies/ml.

Vaccine virus shedding samples with RNA genome levels greater than 5.0 log_10_ PFU/ml were subjected to sequence analysis of the inserted RSV F gene and its flanking sequences to evaluate stability of the vaccine insert. Viral RNA extracted from nasal swabs was amplified by RT-PCR (OneTaq One-Step RT-PCR, New England BioLabs) and cDNA sequenced by the Sanger method (Azenta).

### Immunogenicity assessment

Blood and nasal samples were collected at baseline (day 1, before vaccination), day 15, and day 29 before vaccination. To reduce sample variation, samples from the same participant were run on the same plate in a blinded fashion in all the assays. Serum RSV nAb levels were determined by a qualified RSV A2 microneutralization (MN) assay based on RSV-A2-rLuc reporter virus (*45*). Briefly, serially twofold diluted serum samples in quadruplicate from a starting dilution of 1:100 were incubated with 175 ± 75 PFU RSV-rLuc for 1 hour and infected Vero cells on 96-well, white-walled, clear bottom plates. After 20 to 24 hours incubation, the cells were lysed using Renilla-Glo Luciferase assay (Promega) and luciferase signals were read on a SpectraMax iD3 multimode microplate reader (Molecular Devices). RSV nAb titer was defined as reciprocal dilution that inhibited at least 50% signal of the virus control as determined by 5PL curve fitting using Prism (version 9.5.1 for macOS, GraphPad Software). The RSV nAb titer was converted to international units based on the standard serum (16/284) obtained from The National Institute for Biological Standard and Control (NIBSC) (London, UK).

RSV F–specific serum IgG and IgA Ab and nasal IgA Ab levels were determined by enzyme-linked immunosorbent assay (ELISA) assay using purified RSV F protein (25 ng per well; SinoBiological, catalog no. 11049-V08B) and twofold serial dilutions in blocking buffer (5% milk/0.5% bovine serum albumin in 1× KPL wash buffer (SeraCare) in duplicate. End-point titer was calculated by 4PL curve fitting using Prism and reported as reciprocal dilution. PIV5-specific IgG and nAb titers were determined by ELISA assay using PIV5 virus-coated plates and by PIV5-rLuc–based MN assay in Vero cells, respectively. The PIV5 IgG end-point titer was calculated by 4PL curve fitting and reported as reciprocal dilution. The PIV5 MN assay was performed similarly to the RSV-rLuc–based MN assay, except in duplicate instead of quadruplicate. Analysis of PIV5 MN was identical to the RSV-rLuc–based MN assay, and the nAb titer was defined as the reciprocal dilution that inhibited at least 50% signal of the virus control.

Antigen-specific T cell frequencies were evaluated by intracellular cytokine staining assay using cryopreserved peripheral blood mononuclear cells (PBMCs) isolated from whole blood on day 1 (pre-vaccine dosing), day 15, and day 29. One million cryopreserved PBMCs were thawed in complete 10% fetal bovine serum (FBS) RPMI medium and rested overnight at 37°C before being incubated with an RSV F peptide pool from GenScript at a final concentration of 1 μg/ml in the presence of anti-CD28 ECD (1 μg/ml; Beckman Coulter, clone CD28.2), anti-CD107a fluorescein isothiocyanate (BD Biosciences, clone H4A3), and anti-CD49d (BD Biosciences, clone 9F10). In addition, PBMCs were stimulated with 1 μl of complete medium with 0.5% dimethyl sulfoxide (DMSO; negative control corresponding to the DMSO concentration of the RSV F peptide pool) or 1 μl of phorbol 12-myristate 13-acetate (PMA)/ionomycin [PMA (25 ng/ml) and ionomycin (1 μg/ml)] for negative and positive controls, respectively. After 2-hour incubation at 37°C, brefeldin A (10 μg/ml; BD Biosciences) were added and the cells were incubated for another 4 hours. The cells were washed with phosphate-buffered saline (PBS) and incubated with aqua viability dye (Invitrogen) at room temperature for 15 min. The cells were washed with PBS supplemented with 2% FBS and surface-stained at 4°C for 30 min with anti-CD3 Alexa 700 (BD Biosciences, clone SP34-2), anti-CD4 BV605 (BD Biosciences, clone L200), anti-CD8 BV450 (BD Biosciences, clone RPA-T8), and anti-CD95 phycoerythrin (PE)–Cy5 (BD Biosciences, clone DX2). The cells were washed with PBS with 2% FBS, fixed with Cytofix/Cytoperm (BD Biosciences), permeabilized with 1× Perm/Wash (BD Biosciences), and incubated with anti–IFN-γ PE-Cy7 (BD Biosciences, clone B27), anti–TNF-α allophycocyanin (APC)–Cy7 (BioLegend, clone Mab11), anti–IL-13 PE (Miltenyi Biotec, clone JES10-5A2.2), and anti–MIP-1β APC (eBioscience, clone FL34Z3L) antibodies at 4°C for 30 min. The cells were washed with 1× Perm/Wash and PBS with 2% FBS then resuspended in PBS/2% formaldehyde for acquisition on a BD FACSAria Fusion cell sorter. CD3^+^ cells were gated for CD4^+^ and CD8^+^ T cells and separated into memory and naïve cells with CD28 and CD95. The net percentage of cytokine-secreting cells was determined by subtraction of the values obtained with DMSO-stimulated samples (negative control). T cell frequencies were considered positive if the detected frequency of cytokine-positive CD4^+^ or CD8^+^ T cells was >0.1% after subtraction of the baseline. Data were analyzed using FlowJo software (version 10). A Boolean combination and SPICE software were used to determine polyfunctional responses of CD4^+^ and CD8^+^ T cells producing two or more cytokines.

### Statistical analysis

Statistical analysis was performed using GraphPad Prism software (Version 9). Given the small sample size, statistical analysis was mostly descriptive and summative. *P* values were used to show difference of potential significance at a 0.05 significance level. Two-group comparisons of RSV-specific CMI responses and Ab responses were evaluated by the Wilcoxon matched-pairs signed rank test (day 15 or day 29 versus day 1 or day 15 versus day 29). Antibody titer rises of ≥1.5-fold after vaccination versus baseline was considered significant because assay characteristics showed a change of 1.2- to 1.3-fold (95% confidence) to be a significant change when samples from a single participant were tested on the same plate.
