# Supplementary material for: Intranasal parainfluenza virus type 5 (PIV5)–vectored RSV vaccine is safe and immunogenic in healthy adults in a phase 1 clinical study
Source: Sci Adv. 2023 Oct 25;9(43):eadj7611. doi: 10.1126/sciadv.adj7611 (PMC10599610; doi:10.1126/sciadv.adj7611)
Supplement: Supplementary file 1 — Figs. S1 and S2 [file sciadv.adj7611_sm.pdf]

Science Advances
AAAS

Supplementary Materials for

Intranasal parainfluenza virus type 5 (PIV5)–vectored RSV vaccine is safe and immunogenic in healthy adults in a phase 1 clinical study

Paul Spearman *et al.*

Corresponding author: Biao He, biaohe@bluelakebiotechnology.com; Paul Spearman, paul.spearman@cchmc.org

Sci. Adv. **9**, eadj7611 (2023)
DOI: 10.1126/sciadv.adj7611

This PDF file includes:

Figs. S1 and S2

Supplement Figure 1

A. Individual's RSV nAb response

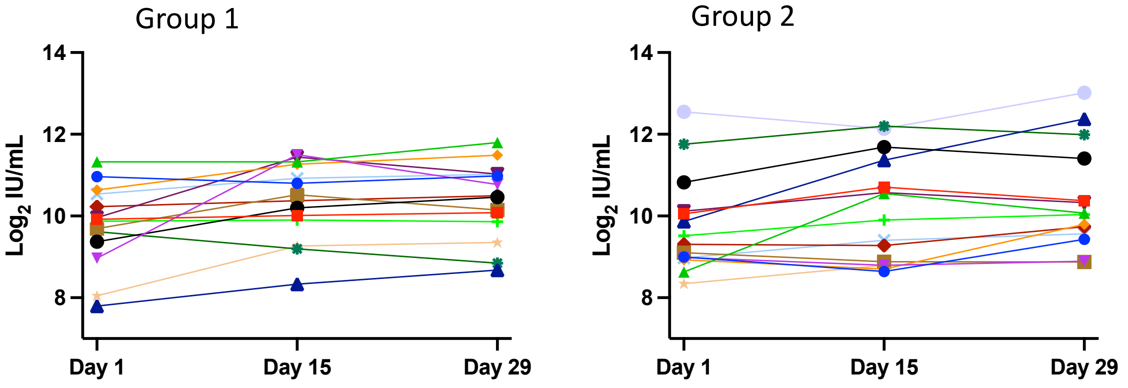

B. Individual's RSV nasal IgA Ab response

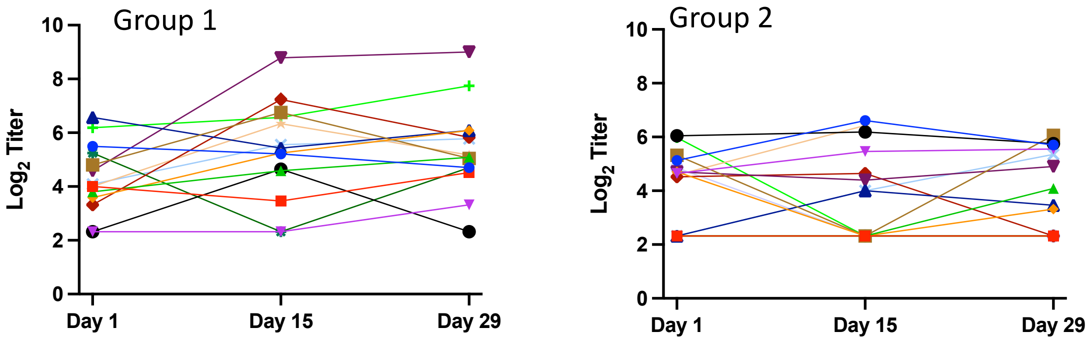

Supplemental Fig 1. (A) Individual serum RSV neutralizing (nAb) titers and (B) nasal F-specific IgA antibody (Ab) titers before and after vaccination (at Days 1, 15 and 29) and by age group.

Supplement Figure 2

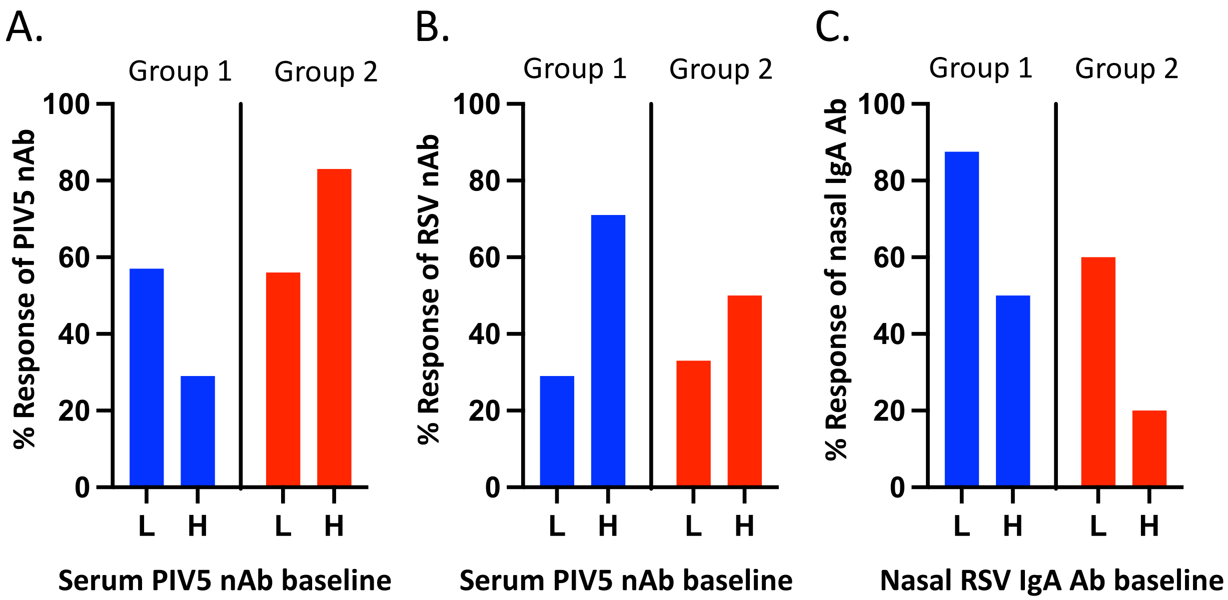

Supplemental Fig 2. Relationships between response rates to BLB201 vaccination and antibody titers at baseline (pre-vaccination). Percentage of subjects with (A) PIV5 neutralizing (nAb) seroresponses to BLB201 (≥1.5-fold rise in titer) and (B) RSV neutralizing (nAb) seroresponses to BLB201 (≥1.5-fold rise in titer) by subgroups based on individual baseline PIV5 nAb titer being below (L) or above (H) the baseline geometric mean titer for PIV5 nAbs for each age group (Group 1 and Group 2). (C) Percentage of subjects with nasal F-specific IgA responses to BLB201(≥2-fold rise in titer) by subgroups based on individual baseline nasal F-specific IgA titer being below (L) or above (H) the baseline geometric mean titer for nasal F-specific IgA for each age group (Group 1 and Group 2).
